# Antimalarial Activity of *Tinospora baenzigeri* against *Plasmodium berghei-*Infected Mice

**DOI:** 10.1155/2019/5464519

**Published:** 2019-09-05

**Authors:** Sakaewan Ounjaijean, Manas Kotepui, Voravuth Somsak

**Affiliations:** ^1^Nutrition Research Unit, Center for Applied Health Sciences Research, Research Institute for Health Sciences, Chiang Mai University, Chiang Mai 50200, Thailand; ^2^School of Allied Health Sciences, Walailak University, Nakhon Si Thammarat 80161, Thailand; ^3^Faculty of Medical Technology, Western University, Kanchanaburi 71170, Thailand

## Abstract

Plant species of the genus *Tinospora* (Menispermaceae) possess several pharmacological properties, and *T. crispa* has been reported to have antimalarial activity. *T. baenzigeri* (Chingcha Chalee) is a rich source of terpenes and quinoline alkaloids; however, it still has not yet been investigated the antimalarial activity of this plant extract. Hence, this study was aimed to evaluate the antimalarial activity of *T. baenzigeri* stem extract against *Plasmodium berghei-*infected mice. The aqueous crude extract of *T. baenzigeri* stem was prepared using a microwave-assisted method and tested for acute toxicity in mice. For evaluating the antimalarial activity *in vivo*, the standard 4-day test was carried out using groups of ICR mice infected with *P. berghei* ANKA administered orally by gavage with the extract (100, 250, and 500 mg/kg) for 4 consecutive days. Parasitemia, body weight, packed cell volume, and mean survival time were then measured. It was found that the aqueous crude extract of *T. baenzigeri* stem did not exhibit any sign of toxicity up to the dose of 2,000 mg/kg. The extract significantly (*P* < 0.01) inhibited parasitemia in a dose-dependent manner, with 22.02%, 50.81%, and 74.95% inhibition. Moreover, the marked prevention of body weight loss and packed cell volume reduction was observed at doses of 100, 250, and 500 mg/kg of extract-treated mice. Additionally, the extract prolonged the mean survival time of *P. berghei-*infected mice, compared to the untreated group. In conclusion, the aqueous crude extract of *T. baenzigeri* stem has demonstrated potent antimalarial activity against *P. berghei-*infected mice with prolonged mean survival time and prevention of body weight loss and packed cell volume reduction.

## 1. Introduction

Malaria is a life-threatening disease caused by protozoan parasites of the genus *Plasmodium* and transmitted by *Anopheles* mosquito. This disease is still a public health problem to humans and the primary cause of morbidity and mortality in the endemic areas [1]. It was estimated in 2016 that ∼216 million cases and ∼445,000 malarial deaths had occurred in the world annually [2]. The incidence of malaria is increasing due to several factors, especially the resistance of the malaria parasite to available antimalarial drugs [3]. Therefore, there is an urgent need to develop and find new drugs for the treatment of malaria. The classical antimalarial drugs, quinine and artemisinin, are both plant derivatives obtained from *Cinchona* species and *Artemisia annua*, respectively, suggesting that other effective antimalarial drugs might be plant-derived [4]. In the current resistance problem, plant materials and extracts are a potential source of new, effective, and affordable antimalarial agents.

Plant species of the genus *Tinospora* (Menispermaceae) possess pharmacological properties such as antioxidant, anti-inflammatory, anticancer, antidiabetic, antimicrobial, and immunostimulatory activities [5, 6]. However, rare attempts were reported on activities of *T. baenzigeri*. *T. baenzigeri* Forman (Thai name: Chingcha Chalee) has been widely distributed throughout the tropical and subtropical areas of Africa, Australia, and Asia [5]. This plant is a woody climber with stem warty and bitter, simple with cordate leaves, and small flowers (information from the Sireeruckhachati Nature Learning Park by Mahidol University). It has been commonly used as a domestic folk medicine for the treatment of headache, cold, fever, diarrhea, ulcer, digestive disorder, and rheumatoid arthritis [7]. It has been reported that only *T. crispa* stem extract presented antimalarial activity against *P. berghei* and *P. yoelii* [8, 9]. The previous research showed that *T. baenzigeri* is a rich source of terpenes and quinoline alkaloids [7]; however, it still has not yet been investigated the antimalarial activity of this plant extract. This study, therefore, aimed to assess the antimalarial activity of the aqueous crude extract of *T. baenzigeri* stem against *P. berghei*-infected mice.

## 2. Materials and Methods

### 2.1. Chemicals and Drug Preparation

In this study, chloroquine (CQ) and Tween-80 purchased from Sigma (Sigma, Chemical, St. Louis, MO, USA) were used. All reagents were of analytical grade. CQ at a chosen dose was freshly prepared in 20% Tween-80.

### 2.2. Plant Material and Extraction

**Figure 1:**
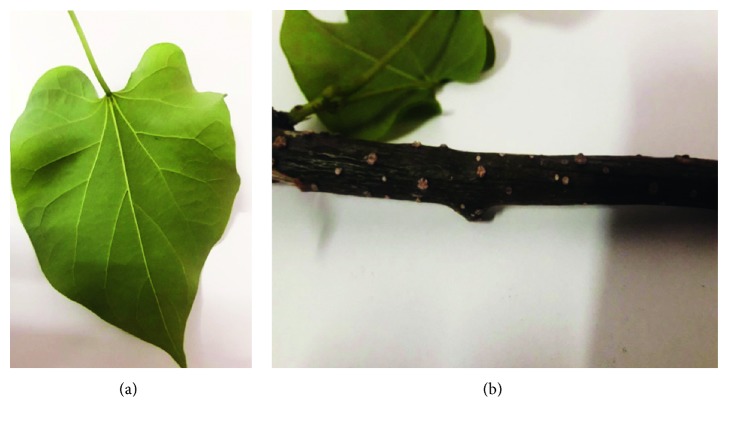
*Tinospora baenzigeri* Forman. (a) Leaf and (b) stem.

Fresh stems of *Tinospora baenzigeri* Forman were collected at Kanchanaburi Province, Thailand (Figure 1). The plant was then identified by Mrs. Jaree Bansiddhi at the Herbarium, the Division of Medicinal Plant Research and Development, Department of Medical Science, Nonthaburi 11000, Thailand, and a voucher specimen (CC09) was deposited at the Faculty of Medical Technology, Western University. The stems of *T. baenzigeri* were cleaned and air-dried in the shade at room temperature. The dried stems were powdered using an electric blender and subsequently stored at room temperature until extraction.

For extraction, the powdered plant material was extracted with distilled water at the ratio of 1 : 10 using a microwave-assisted method [10, 11] and incubated at room temperature for 3 consecutive days with occasional stirring. After that, filtration was carried out with the Whatman no. 1 filter paper, and the filtrate was then dried by lyophilization. The aqueous crude extract of *T. baenzigeri* stem was kept at −20°C until further use. Before the experiment, the extract was dissolved in 20% Tween-80 at appropriate doses for oral administration in mice.

### 2.3. Animals and Rodent Malaria Parasites

We obtained the female ICR mice, 4–6 weeks of age, weighing between 25 and 35 g, from the National Laboratory Animal Center, Mahidol University, Thailand. Standard pellet diet and clean water *ad libitum* were fed to the mice housed in standard cages at a temperature of 25 ± 2°C with a 12 h photoperiod/day. The Ethics Committee of Western University approved to perform the experiment (WTU/AEC002/2017).

In this study, the chloroquine-sensitive *Plasmodium berghei* ANKA strain (PbANKA) obtained from the Malaria Research and Reference Reagent Resource Center (MR4; http://www.malaria.mr4.org/) was used. The parasites were maintained weekly by serial passage of blood from the donor-infected mice to naïve mice via intraperitoneal (IP) injection. Propagation of the parasite *in vivo* was monitored daily by microscopic analysis of Giemsa-stained thin blood smears. Parasitemia was calculated as follows:

(1) $$\begin{array}{c} \text{\% parasitemia }=\;\frac{\text{number of parasitized erythrocytes}}{\text{total number of erythrocytes}}\;\times \;100\text{.} \end{array}$$

### 2.4. Acute Toxicity Test of the Crude Extract in Mice

The acute toxicity test of the aqueous crude extract of *T. baenzigeri* stem was carried out using the method described previously [12]. Before the experiment, the ICR mice were starved for 3-4 hr with only water allowed and 1-2 h after the administration of the extracts. Twenty ICR mice were divided randomly into 4 groups (5 mice of each) and were administered orally by gavage increasing doses of 500, 1,000, and 2,000 mg/kg of the aqueous crude extract of *T. baenzigeri* stem. The control group received 0.2 ml of the respective vehicle (20% Tween-80). Next, these mice were observed for 1 hr and a period of 24 hr for behavioral changes such as sleep, rigidity, mortality, and other signs of acute toxicity and were followed for 28 days. Moreover, liver and kidney injury parameters including aspartate aminotransferase (AST), alanine aminotransferase (ALT), blood urea nitrogen (BUN), and creatinine (Cre) were also measured using a Cobas c311 automate analyzer from mouse blood collected by cardiac puncture.

### 2.5. Efficacy of Antimalarial Screening In Vivo

*Plasmodium berghei* 4-day suppressive test was carried out by a method described previously [13]. After standard parasite inoculation of 1 × 10^7^ parasitized erythrocytes of PbANKA by IP injection, 25 mice were divided randomly into 5 groups (5 mice each). Group I mice were treated with 10 ml/kg of the vehicle (20% Tween-80) as untreated control, group II, III, and IV mice were treated with 100, 250, and 500 mg/kg of the extract, respectively, and group V mice were treated with 10 mg/kg of CQ. Treatment was started 2 hours after infection and continued for 4 consecutive days (D0 to D3) via orally by gavage. On D4, parasitemia was determined by microscopic analysis of Giemsa-stained thin blood smears, and the percentage of inhibition was subsequently calculated using the following formula:

(2) $$\begin{array}{c} \text{\% inhibition }=\;\frac{\left(\text{\% parasitemia of untreated control}-\text{\% parasitemia of treated group}\right)\;}{\text{ \% parasitemia of untreated control}}\;\times \;100. \end{array}$$

### 2.6. Determination of Body Weight and Packed Cell Volume

The body weight (BW) and packed cell volume (PCV) of each mouse were determined before and after infection. The average BW was calculated as follows:

(3) $$\begin{array}{c} \text{BW }=\;\frac{\text{total BW of mice in a group}}{\text{total number of mice in that group}}\text{.} \end{array}$$

The tail blood of each mouse was collected into the heparinized microhematocrit tubes up to 3/4^th^ of their length for PCV measurement. The tubes were sealed by crystal seal and then centrifuged at 12,000 rpm for 4 min. The PCV was calculated as follows:

(4) $$\begin{array}{c} \text{PCV }=\;\frac{\text{volume of erythrocytes in a given volume of blood}}{\text{total blood volume}}\;\times \;100. \end{array}$$

### 2.7. Determination of Mean Survival Time

Mortality for each mouse in the treatment and control groups was daily observed as the number of days from the time of inoculation up to death throughout the follow-up period. The mean survival time (MST) for each group was subsequently calculated as

(5) $$\begin{array}{c} \text{MST }=\;\frac{\text{sum of survival time of all mice in a group}\left(\text{days}\right)}{\text{total number of mice in that group}}. \end{array}$$

### 2.8. Statistical Analysis

Results of this study were presented as the mean ± standard error of mean (SEM). The data obtained from parasitemia, BW, PCV, and MST were analyzed using GraphPad Prism (GraphPad Prism Software, Inc., USA). The one-way ANOVA (analysis of variance) followed by Tukey's post hoc test was used to compare the differences between means of the measured parameters. Statistical significance was considered at 95% confidence level, *P* values <0.05.

## 3. Results

### 3.1. Acute Toxicity of *T. baenzigeri* Stem Extract in Mice

The mice administered orally by gavage in a single dose up to 2,000 mg/kg of the aqueous crude extract of *T. baenzigeri* stem showed no mortality effect within 24 h of observation. Moreover, physical and behavioral observations of these mice presented no visible signs of toxicity such as hair erection, urination, lacrimation, weight loss, diarrhea, depression, and reduction in feeding and motor activities. No fatalities occurred within the observation period of 28 days. In addition, liver and kidney injuries were also ruled out (Table 1). Hence, the LD50 is greater than 2,000 mg/kg.

### 3.2. Antimalarial Activity of *T. baenzigeri* Stem Extract against PbANKA-Infected Mice

**Figure 2:**
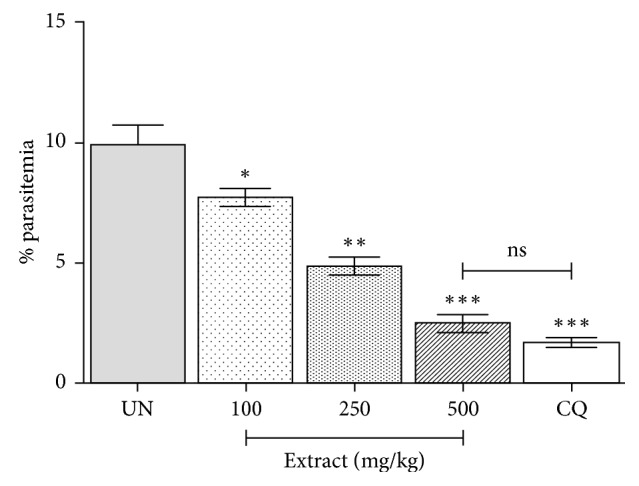
Antimalarial activity of *Tinospora baenzigeri* against *Plasmodium berghei*-infected mice. Groups of mice (5 mice of each) were inoculated with 1 × 10^7^ parasitized erythrocytes of PbANKA by IP injection. They were then administered orally by gavage with 100, 250, and 500 mg/kg of extract once a day for 4 consecutive days. Parasitemia was then determined by microscopic analysis of Giemsa-stained thin blood smear. ^*∗*^*P* < 0.05, ^*∗∗*^*P* < 0.01, and ^*∗∗∗*^*P* < 0.001, compared to the untreated group. UN, untreated group; CQ, 10 mg/kg of chloroquine; ns, not significant. Results shown (mean ± SEM; *n* = 5 per group) were representative of 2 independent experiments.

The results of this study indicated that the aqueous crude extract of *T. baenzigeri* stem exerted a potent antimalarial activity against PbANKA in a dose-dependent manner (Figure 2). The significant (*P* < 0.05) inhibition of parasitemia produced by the extract was 22.02%, 50.81%, and 74.95% at the dose levels of 100, 250, and 500 mg/kg, respectively. Moreover, the mice treated with CQ (10 mg/kg) caused suppression of 82.83%, which was higher than those of the extract-treated groups. Interestingly, the extract at a dose of 500 mg/kg did not show statistically significant difference as compared to the CQ-treated group.

### 3.3. Effect of *T. baenzigeri* Stem Extract on BW and PCV in PbANKA-Infected Mice

The aqueous crude extract of *T. baenzigeri* stem has markedly prevented malaria-related BW loss, and the mean BW between D0 and D4 in mice treated with the extract at all 3 dose levels did not show statistically significant difference as compared to the respective untreated group (Table 2). Moreover, the result showed a significant (*P* < 0.001) reduction in PCV between D0 and D4 in the respective untreated group. However, mice treated with the extract did not show significant PCV reduction (Table 3).

### 3.4. Effect of *T. baenzigeri* Stem Extract on MST in PbANKA-Infected Mice

As shown in Table 4, the MST of the aqueous crude extract of *T. baenzigeri* stem was significantly (*P* < 0.01) increased, compared to the respective untreated group, and it was prolonged in a dose-dependent manner. Interestingly, the 500 mg/kg of the extract showed markedly prolonged MST similar to the CQ-treated group.

## 4. Discussion

The antimalarial activity of the aqueous crude extract of *T. baenzigeri* stem against *P. berghei-*infected mice was reported in this study. The extract did not cause mortality or any sign of acute toxicity in the mice after oral administration of the extract at doses of 500 to 2,000 mg/kg. If a test substance has a lethal dose (LD50) higher than 3 times the minimum effective dose (100 mg/kg), it can be a good candidate for further studies [14]. Hence, the aqueous crude extract of *T. baenzigeri* stem can be considered safe according to the OECD (Organization for Economic Co-operation and Development) guideline, which recommends a maximum dose of 2,000 mg/kg for acute toxicity [15], and this could explain the use of this plant for traditional management of malaria.

Significant BW loss in the untreated group was observed. It is well established that BW loss is one manifestation of rodent malaria infection. This is due to the depressant action on the appetite of the mice and disturbed metabolic function and the hypoglycemic effect of the malaria parasite [16]. Interestingly, the prevention of BW loss among PbANKA-infected mice after administering 100, 250, and 500 mg/kg of the extract suggested the effect of this extract in preventing malaria-related BW loss. It could be suggested that the extract possesses the activity which might lead to preventing BW loss, including stimulation of appetite and nutritionally endowing with vitamin B1, B2, and B3 [17, 18].

The influence of malaria on PCV reduction is considered as a hallmark of both human and rodent malaria infection. A significant reduction of PCV was observed in the untreated group. These infected mice may suffer from severe anemia because of rapid hemolysis by parasitemia. In addition, erythrocyte fragility was increased during *P. berghei* infection and led to subsequent PCV reduction [19]. However, the absence of a significant reduction of PCV among extract-treated groups at the doses of 100, 250, and 500 mg/kg may indicate the prevention of malaria-related PCV reduction of the crude extract.

The 4-day test is a standard antimalarial screening test *in vivo* in which ≥30% inhibition in parasitemia following treatment makes the extract to be considered active [14]. Accordingly, the aqueous crude extract of *T. baenzigeri* stem which presented 22.02%, 50.81%, and 74.95% inhibition at the doses of 100, 250, and 500 mg/kg, respectively, can be classified as active. This can be explained by the fact that this antimalarial activity of the extract might be attributed to the presence of bioactive secondary metabolites such as phenols, flavonoids, glycosides, tannin, terpenes, and alkaloids [4, 20]. These compounds have been reported to have antimalarial activity [21]. Moreover, isoquinoline alkaloids, clerodane diterpenes, and baenzigeroside might also have antimalarial activity [5, 7]. However, these compounds need to be confirmed in future studies. The possible mechanisms of antimalarial activity might be through free radical scavenging, immunomodulatory, interference of protein synthesis, and inhibition of parasite invasion of new RBCs [22–24].

Accordingly, the extract that can prolong the MST of infected mice compared to the untreated group is considered as active [25]. In this study, the PbANKA-infected mice treated with 100, 250, and 500 mg of the extract had significantly lived longer than the untreated group. The extract at a dose of 500 mg/kg presented the highest MST similar to the CQ-treated group. This might be due to the antimalarial activity of the extract.

## 5. Conclusion

From this study, it can be concluded that the aqueous crude extract of *T. baenzigeri* stem exerted active antimalarial activity against *P. berghei-*infected mice through the significant reduction of parasitemia and therefore prolonged the MST. Moreover, the prevention of PCV reduction and BW loss in mice during malaria infection of this extract was also observed. Accordingly, with the essence of further studies, this plant could serve as the potential new antimalarial drug for the treatment of malaria.

## Figures and Tables

**Table 1:** Effect of oral administration of *Tinospora baenzigeri* on liver and kidney injuries in mice.

Parameters	Control	*T. baenzigeri* extract		
	20% Tween-80	500 mg/kg	1,000 mg/kg	2,000 mg/kg
AST (U/L)	32.62 ± 0.47	33.74 ± 0.58	34.47 ± 0.51	34.38 ± 0.49
ALT (U/L)	15.23 ± 0.33	14.71 ± 0.28	15.57 ± 0.32	14.61 ± 0.37
BUN (mg/dl)	42.22 ± 2.71	41.53 ± 3.48	42.61 ± 3.32	43.74 ± 3.14
Cre (mg/dl)	2.02 ± 0.15	2.20 ± 0.12	2.05 ± 0.17	2.11 ± 0.14

Results shown (mean ± SEM; *n* = 5 per group) were representative of 2 independent experiments.

**Table 2:** Effect of *Tinospora baenzigeri* on body weight of *Plasmodium berghei*-infected mice.

Test	Dose	Body weight (D0)	Body weight (D4)	% change
Untreated	10 ml/kg	26.3 ± 1.14	20.6 ± 1.37^*∗∗∗*^	−21.67
Extract	100 mg/kg	24.2 ± 1.31	23.7 ± 1.24	−2.07
	250 mg/kg	24.7 ± 0.25	24.5 ± 0.42	−0.81
	500 mg/kg	25.1 ± 1.18	25.0 ± 1.02	−0.40
CQ	10 mg/kg	24.6 ± 0.84	24.1 ± 1.48	−2.03

^*∗∗∗*^ *P* < 0.001, compared to D0. Results shown (mean ± SEM; *n* = 5 per group) were representative of 2 independent experiments.

**Table 3:** Effect of *Tinospora baenzigeri* on packed cell volume of *Plasmodium berghei-*infected mice.

Test	Dose	% PCV (D0)	% PCV (D4)	% change
Untreated	10 ml/kg	51.7 ± 1.24	40.2 ± 1.47^*∗∗∗*^	−22.24
Extract	100 mg/kg	52.6 ± 1.17	51.2 ± 1.29	−2.66
	250 mg/kg	53.4 ± 1.92	52.7 ± 1.42	−1.31
	500 mg/kg	52.2 ± 1.18	51.9 ± 1.37	−0.57
CQ	10 mg/kg	51.1 ± 1.29	50.9 ± 1.56	−0.39

^*∗∗∗*^ *P* < 0.001, compared to D0. Results shown (mean ± SEM; *n* = 5 per group) were representative of 2 independent experiments.

**Table 4:** Effect of *Tinospora baenzigeri* on mean survival time of *Plasmodium berghei*-infected mice.

Test	Dose	MST (days)
Untreated	10 ml/kg	9.2 ± 1.22
Extract	100 mg/kg	16.7 ± 3.24^*∗∗*,#,b^
	250 mg/kg	23.5 ± 3.16^*∗∗∗*,a^
	500 mg/kg	26.3 ± 3.01^*∗∗∗*^
CQ	10 mg/kg	28.1 ± 2.12^*∗∗∗*^

^*∗∗*^ *P* < 0.01 and ^*∗∗∗*^*P* < 0.001, compared to the untreated group. ^#^*P* < 0.05, compared to 250 mg/kg and 500 mg/kg of extract-treated groups. ^a^*P* < 0.05 and ^b^*P* < 0.01, compared to the CQ-treated group. Results shown (mean ± SEM; *n* = 5 per group) were representative of 2 independent experiments.

## Data Availability

The data used to support the findings of this study are available from the corresponding author upon request.
